# Surgery After Induction Immuno-Chemotherapy in Stage III-N3 Non-Small Cell Lung Cancer: A Single-Centre Retrospective Cohort Study

**DOI:** 10.1093/icvts/ivag166

**Published:** 2026-07-11

**Authors:** Xiaoping Zeng, Qianxin Zhou, Suyu Wang, Lu Han, Peng Zhang, Ziyun Shen, Yiming Zhou, Jing Zhang

**Affiliations:** Department of Thoracic Surgery, Shanghai Pulmonary Hospital, School of Medicine, Tongji University, Shanghai 200092, China; Department of Thoracic Surgery, Shanghai Pulmonary Hospital, School of Medicine, Tongji University, Shanghai 200092, China; Department of Thoracic Surgery, Shanghai Pulmonary Hospital, School of Medicine, Tongji University, Shanghai 200092, China; Department of Thoracic Surgery, Shanghai Pulmonary Hospital, School of Medicine, Tongji University, Shanghai 200092, China; Department of Thoracic Surgery, Shanghai Pulmonary Hospital, School of Medicine, Tongji University, Shanghai 200092, China; Department of Thoracic Surgery, Shanghai Pulmonary Hospital, School of Medicine, Tongji University, Shanghai 200092, China; Department of Thoracic Surgery, Shanghai Pulmonary Hospital, School of Medicine, Tongji University, Shanghai 200092, China; Department of Thoracic Surgery, Shanghai Pulmonary Hospital, School of Medicine, Tongji University, Shanghai 200092, China

**Keywords:** immuno-chemotherapy, neoadjuvant therapy, N3, non-small cell lung cancer, surgical resection

## Abstract

**Objectives:**

The goal of this study was to preliminarily evaluate the efficacy of a surgical procedure following induction immuno-chemotherapy and nodal downstaging in patients with stage III-N3 non-small cell lung cancer (NSCLC).

**Methods:**

A retrospective analysis was conducted on patients with stage III-N3 NSCLC who received induction immuno-chemotherapy at Shanghai Pulmonary Hospital from August 2019 to May 2023. Patients who achieved confirmed N3 lymph node downstaging, had resectable tumours, and maintained adequate performance status subsequently underwent a surgical procedure after induction therapy. In this post-induction comparative analysis, baseline characteristics and oncological outcomes were compared between patients who underwent surgical procedures and those who did not.

**Results:**

After induction therapy, 28 (24.6%) patients underwent a surgical procedure, while 78 (73.6%) did not. The patients in the surgical group were younger (*P *= .012), had a higher body mass index (*P *= .032), and exhibited a higher proportion of contralateral mediastinal or hilar lymph node involvement (*P *= .024). Compared with those who did not undergo a surgical procedure, patients who did undergo a surgical procedure showed better progression-free survival (hazard ratio [HR] = 0.46, 95% CI, 0.23-0.92) and overall survival (HR = 0.31, 95% CI, 0.11-0.88). However, in the biopsy subgroup, patients who underwent a surgical procedure had better overall survival (HR = 0.64, 95% CI, 0.19-2.20) but similar progression-free survival (HR = 0.97, 95% CI, 0.42-2.23) compared with those who did not undergo a surgical procedure.

**Conclusions:**

These real-world findings provide early positive signals that a surgical procedure after induction immuno-chemotherapy may be feasible in carefully selected patients with stage III-N3 NSCLC.

## Graphical abstract

**Figure:**
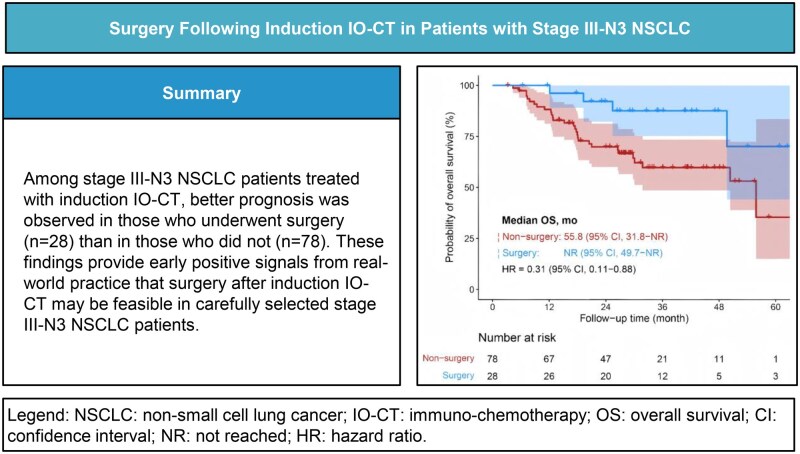

## INTRODUCTION

Patients with stage III-N3 non-small cell lung cancer (NSCLC) are generally considered unresectable, with a median survival time of less than 19 months and a 5-year survival rate of approximately 20%.^1^ Definitive concurrent chemoradiotherapy (cCRT) is the standard initial treatment for N3 disease.^2^ The PACIFIC trial has demonstrated that consolidation immunotherapy following cCRT significantly improves long-term survival in unresectable stage III NSCLC patients.^3^ However, the benefits of immunotherapy are limited to those without progression after cCRT, as underscored by the failure of immunotherapy combined with cCRT based on the results of the PACIFIC-2 study (NCT03519971).

Neoadjuvant immuno-chemotherapy has markedly improved R0 resection and pathological complete response (pCR) rates in resectable patients with NSCLC compared to chemotherapy alone.^4–6^ The substantial tumour shrinkage rates observed with immunotherapy have driven investigations into converting initially unresectable tumours to resectable ones. Recent trials have indicated that induction immuno-chemotherapy can downstage some initially unresectable stage III NSCLCs, permitting surgical resection with favourable long-term survival outcomes.^7–10^

Notably, patients with stage III-N3 NSCLC have been reported to achieve downstaging and have subsequently undergone a surgical procedure following induction immuno-chemotherapy in a few cases.^11–13^ A nationwide study has also suggested potential long-term survival benefits of a surgical procedure in selected N3 patients.^14^ Nevertheless, further evidence is needed to support the efficacy of a surgical procedure following induction immuno-chemotherapy in patients with stage III-N3 NSCLC in a real-world setting. Therefore, we conducted a retrospective study to preliminarily evaluate the role of a surgical procedure following induction immuno-chemotherapy in patients with stage III-N3 NSCLC.

## METHODS

### Study cohort

This retrospective study was approved by the Ethics Committee of Shanghai Pulmonary Hospital and was conducted in accordance with the Declaration of Helsinki (approval ID: K24-685). This study did not involve biological materials or biobank resources. All anonymized data were processed in accordance with French data protection laws and the World Medical Association Declaration of Taipei. The requirement for written informed consent was waived due to the retrospective design.

Patients with clinical stage N3 NSCLC who received immuno-chemotherapy between August 2019 and May 2023 at the Shanghai Pulmonary Hospital were enrolled retrospectively. Exclusion criteria included (1) lack of confirmation by positron emission tomography-computed tomography (PET/CT) or biopsy; (2) a prior history of cancer-specific treatment; (3) combined small cell lung cancer; (4) distant metastasis at initial diagnosis; and (5) presence of a mediastinal mass. Patients were divided into the surgical procedure group and the non-surgical procedure group based on whether they underwent a surgical procedure after induction therapy. Tumours were restaged according to the 9th edition of the TNM staging for lung cancer.^1^^,^^15^

### Induction therapy and response evaluation

Patients received a standardized regimen of programmed death receptor 1 (PD-1) blockades combined with platinum-doublet chemotherapy, routinely administered in treatment cycles at our centre. Radiologic response after induction treatment was evaluated by 2 senior radiologists at our centre according to the Response Evaluation Criteria in Solid Tumours version 1.1 (RECIST v1.1).^16^

A major pathological response (MPR) was defined as ≤10% surviving tumour cells, whereas pCR was defined as no residual viable tumour cells.^17^^,^^18^ Pathological responses were independently assessed by 2 experts from the Department of Pathology at our centre.

### Surgical approach

Patients who expressed a strong desire for a surgical procedure were reevaluated for surgical feasibility under the guidance of the multidisciplinary team. The criteria for surgical eligibility included (1) confirmation of N3 lymph node downstaging after induction therapy using methods consistent with those used in the initial evaluation; (2) resectability of the primary tumour; and (3) sufficient performance status to tolerate a surgical procedure.

### Study outcomes

Progression-free survival (PFS) was defined as the time interval from initiation of induction treatment to disease progression (according to RECIST v1.1) or death from any cause. Overall survival (OS) was defined as the duration from initiation of induction treatment to death from any cause. Follow-up was conducted via outpatient records or telephone interviews, with the last follow-up on 15 July 2025.

### Statistical analysis

Normally distributed continuous variables are presented as mean and stable disease, whereas non-normally distributed data are expressed as median and interquartile range. The 2-sample *t*-test or the Mann–Whitney *U*-test was used for analysis. Categorical data are reported as frequencies and percentages, compared using the χ^2^ test or the Fisher exact test. Kaplan–Meier survival curves for PFS and OS were generated. The duration of follow-up was estimated using the inverse Kaplan–Meier method. A landmark analysis at 18 weeks after initiation of induction therapy was conducted to compare the surgical and the non-surgical groups. All tests were 2-tailed, and *P *< .05 was considered to be statistically significant. Data analysis was conducted using the R software (version 4.4.2).

## RESULTS

### Baseline characteristics

**Figure 1.**
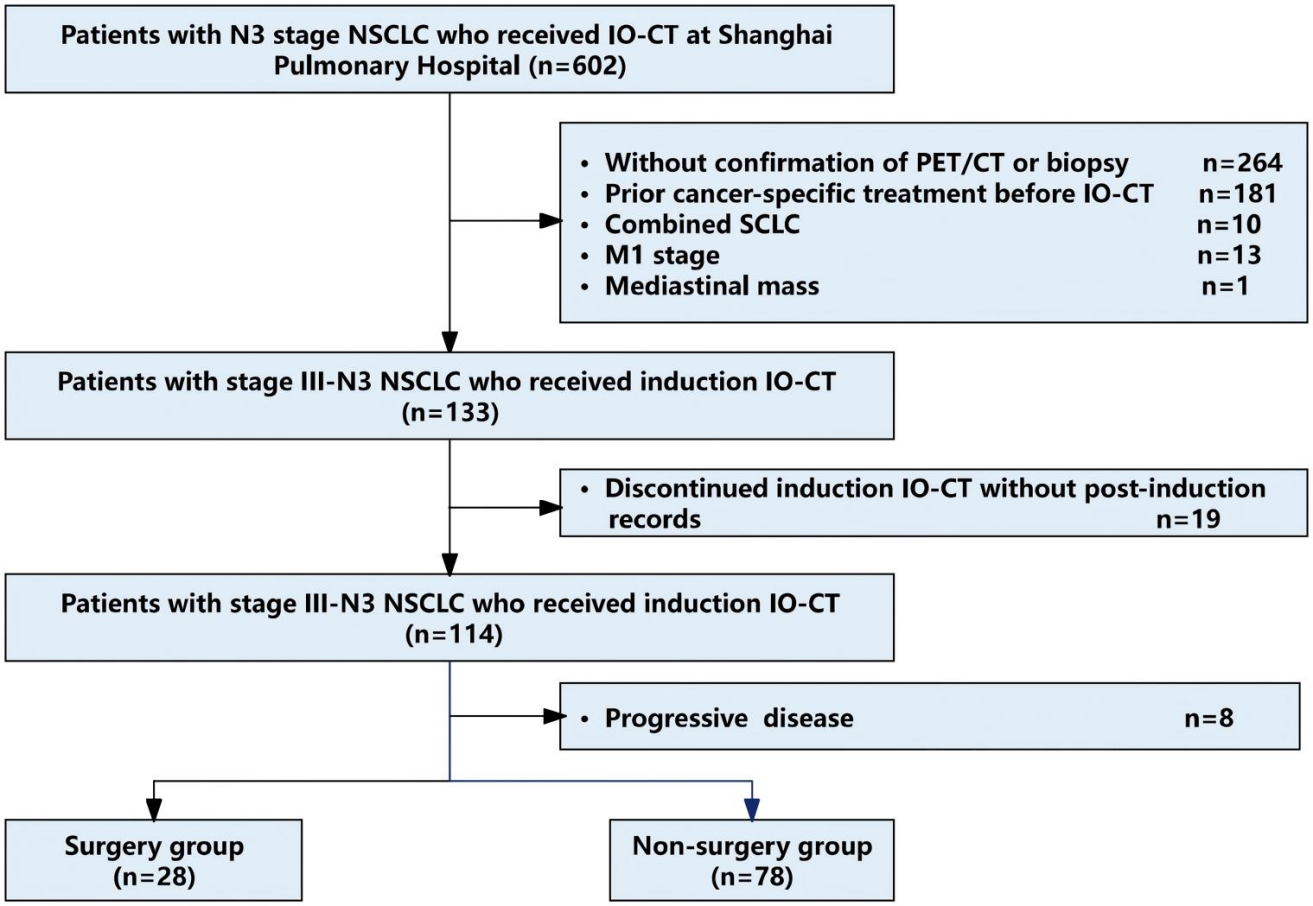
Flow Chart for Patient Selection and Grouping Process. Abbreviations: IO-CT, immuno-chemotherapy; N, node; NSCLC, non-small cell lung cancer; PET, positron emission tomography; SCLC, small cell lung cancer.

A total of 133 patients are included in the analysis (**Figure 1**). The median age was 66 years (IQR: 58-71), and there was a predominance of males (92.5%). N3 lymph node metastasis was confirmed by biopsy in 67 (50.4%) patients; the remaining half were suspected of having N3 lymph node metastasis based on PET/CT scans. Regarding N3 lymph node involvement, 68 (51.1%) patients had supraclavicular lymph node metastasis, 58 (43.6%) had contralateral mediastinal or hilar lymph node metastasis, and 7 (5.3%) had both. Detailed demographics and clinical characteristics are shown in **Table 1**.

**Table 1.** The Demographic and Clinical Characteristics of All Patients

Characteristics	Overall (*n* = 133)
Age (median [IQR], year)	66.00 (58.00, 71.00)
Sex, male, *n* (%)	123 (92.5)
Smoking history, *n* (%)	87 (65.4)
BMI (mean [SD], kg/m^2^)	23.83 (3.20)
ECOG, *n* (%)	
0	5 (3.8)
1	126 (94.7)
2	2 (1.5)
Comorbidity, *n* (%)	
Hypertension	34 (25.6)
Diabetes mellitus	13 (9.8)
Cardiovascular disease	5 (3.8)
Pulmonary disease	16 (12.0)
Tumour location, right, *n* (%)	60 (45.1)
Tumour size before therapy (median [IQR], mm)	45.00 (32.00, 63.00)
Histologic type, *n* (%)	
Squamous cell carcinoma	69 (51.9)
Adenocarcinoma	41 (30.8)
Other	23 (17.3)
cT stage before therapy, *n* (%)	
T1	24 (18.0)
T2	46 (34.6)
T3	31 (23.3)
T4	32 (24.1)
N3 lymph nodes metastasis, *n* (%)	
Supraclavicular	68 (51.1)
Mediastinal or hilar	58 (43.6)
Both	7 (5.3)
Diagnostic methods for N3 lymph nodes, *n* (%)	
Biopsy	67 (50.4)
PET/CT only	66 (49.6)
PD-L1 TPS, *n* (%)	
<1%	34 (25.6)
1-49%	23 (17.3)
≥50%	23 (17.3)
Unknown	53 (39.8)
Cycles of induction therapy, median (IQR)	4.00 (2.00, 4.00)
Clinical response after induction therapy, *n* (%)	
PR	73 (54.9)
SD	33 (24.8)
PD	8 (6.0)
Not evaluable	19 (14.3)

Abbreviations: BMI, body mass index; ECOG, Eastern Cooperative Oncology Group; IQR, interquartile range; PD, progressive disease; PET/CT, positron emission tomography-computed tomography; PR, partial response; SD, stable disease.

### Induction therapy and treatment efficacy

**Figure 2.**
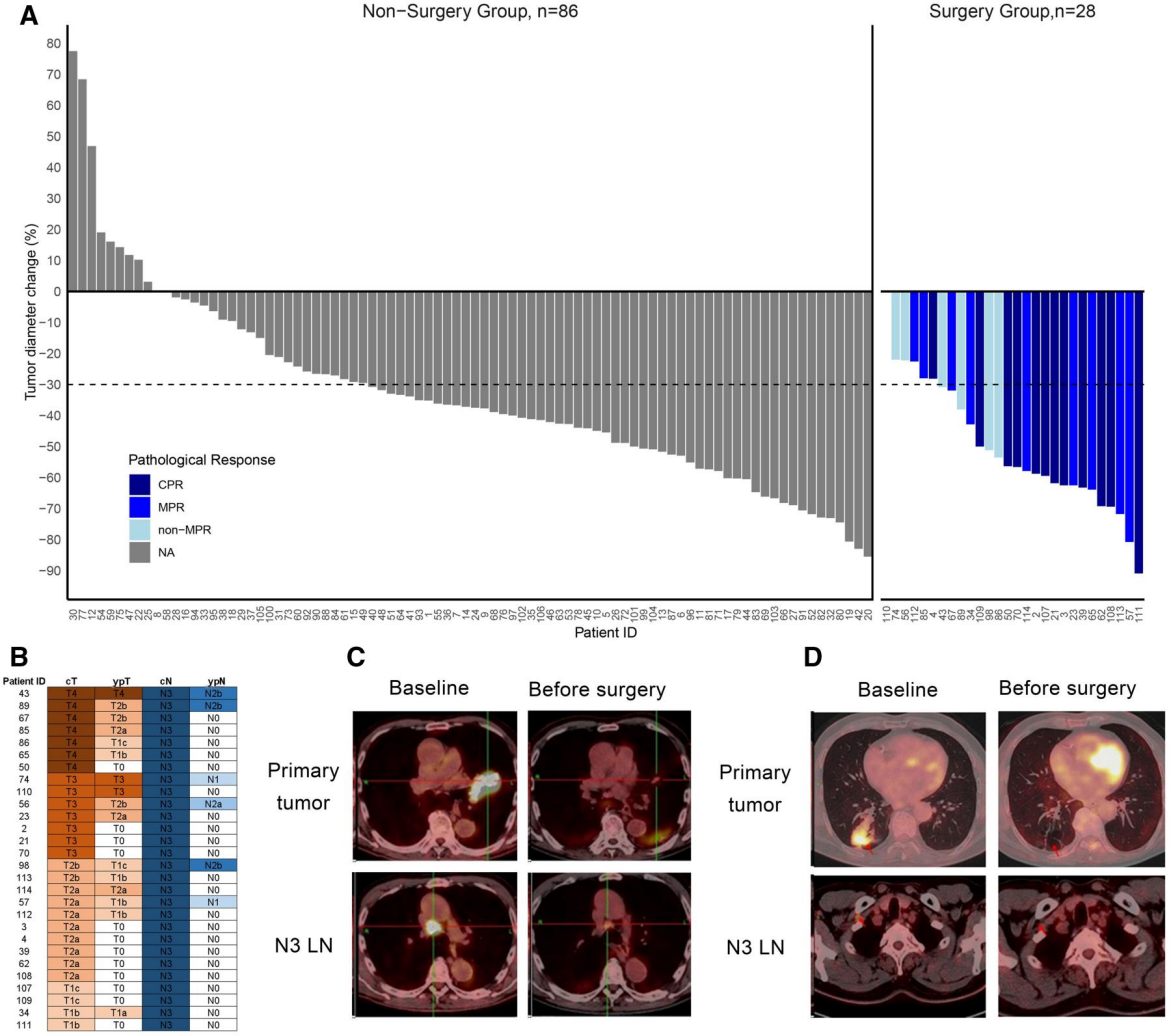
Radiologic and Pathological Responses to Induction Immuno-Chemotherapy. (A) Forest plot for radiologic tumour diameter change. (B) Clinical stage before induction immuno-chemotherapy and pathological stage postoperatively. (C) Typical case with contralateral mediastinal lymph node involvement. (D) Typical case with supraclavicular lymph node involvement. Abbreviations: CPR, complete pathological response; ID, identification; LN, lymph node; MPR, major pathological response; NA, not applicable.

Patients received a median of 4 cycles (IQR: 2-4) of induction therapy. After induction therapy, 73 (54.9%) patients had a partial response, 33 (24.8%) had stable disease, 8 (6.0%) had progressive disease, and 19 (14.3%) were not evaluable due to discontinuation of induction therapy. Radiologic changes evident in the diameter of the tumour and pathological responses are presented in **Figure 2A**. The efficacy of induction therapy stratified by the metastatic site of the N3 lymph node, the type of PD-1 blockade, and the PD-L1 tumour proportion score (TPS) are shown in **Figures S1A-S3A**.

### Surgical information and pathological response

During induction therapy, 19 (14.3%) patients discontinued treatment without any post-induction records. Among the remaining 114 (85.7%) patients, 8 (6.0%) with progressive disease were excluded from surgical evaluation, 60 (45.1%) underwent evaluation for surgical feasibility, and 46 (34.6%) did not. Among the 32 (24.1%) patients who were evaluated but ultimately did not undergo a surgical procedure, the primary reasons were unresectability (*n* = 24, 75.0%), operability issues due to poor physical condition (*n* = 4, 12.5%), and patient refusal to undergo the operation (*n* = 4, 12.5%).

Finally, 28 (21.1%) patients underwent surgical resection. Complete resection of the primary tumour was achieved in all patients, with surgical information summarized in **Table S1**. Of the 28 patients, 15 (53.6%) underwent video-assisted thoracoscopic surgery, 10 (35.7%) underwent thoracotomy, and 3 (10.7%) underwent robotic-assisted thoracoscopic surgery. Twenty-one (75.0%) patients underwent lobectomy; 6 (21.4%) underwent sleeve resection; and 1 (3.6) underwent bilobectomy. Postoperative complications were observed in 15 (53.6%) patients, with pleural effusion (*n* = 9, 32.1%) and prolonged air leak (*n* = 7, 25.0%) being the most frequent. No 30-day postoperative deaths were reported. Only 1 patient underwent the dissection of contralateral mediastinal lymph nodes (4L, 5, and 6) concomitantly with a right lower lobectomy and ipsilateral systematic mediastinal lymph node dissection during the same video-assisted thoracoscopic surgery procedure under the same episode of general anaesthesia. No patient underwent supraclavicular lymph node dissection.

Following the surgical procedure, 21 (75.0%) patients achieved an MPR, including 12 (42.9%) who achieved a pCR. The information on postoperative therapy is summarized in **Table S2**. The clinical and pathological stages of the surgically treated group are shown in **Figure 2B**. The PET/CT images of 2 patients (1 with contralateral mediastinal N3 and 1 with supraclavicular N3) are shown in **Figure 2C and D**. The pathological response status stratified by the metastatic site of the N3 lymph node, the type of PD-1 blockade, and the PD-L1 tumour proportion score is shown in **Figures S1B-S3B**.

### Surgical group vs non-surgical group

Patients who discontinued induction therapy with unknown surgical status or disease condition (*n* = 19), as well as those who experienced disease progression during induction therapy (*n* = 8), were excluded from the comparison between the surgical and the non-surgical groups. Finally, there were 28 (26.4%) patients in the surgical group and 78 (73.6%) patients in the non-surgical group. The information regarding the non-surgical treatments received by the patients in the non-surgical group is summarized in **Table S3**.

The baseline characteristics of the surgical and non-surgical groups are compared in **Table 2**. Patients in the surgical group were significantly younger (*P *= .012), had a higher body mass index (*P *= .032), and had a higher proportion of contralateral mediastinal or hilar lymph node involvement (*P *= .024). There were no significant differences between the 2 groups in other baseline characteristics (all *P *> .05).

**Table 2.** Demographic and Clinical Characteristics of Surgical Compared With Non-Surgical Groups

Characteristics	Non-surgical group (*n* = 78)	Surgical group (*n* = 28)	*P-*value
Age (median [IQR], year)	68.00 (60.00, 72.00)	60.00 (56.50, 67.50)	**.012**
Sex, male, n (%)	73 (93.6)	26 (92.9)	1.000
Smoking history, *n* (%)	49 (62.8)	22 (78.6)	.128
BMI (mean [SD], kg/m^2^)	23.42 (3.11)	24.95 (3.45)	**.032**
ECOG, n (%)^a^			1.000^a^
0	2 (2.6)	1 (3.6)	
1	75 (96.2)	27 (96.4)	
2	1 (1.2)	0 (0)	
Hypertension, *n* (%)	17 (21.8)	9 (32.1)	.275
Diabetes mellitus, *n* (%)	10 (12.8)	2 (7.1)	.641
Cardiovascular disease, *n* (%)	5 (6.4)	0 (0)	.394
Pulmonary disease, *n* (%)	8 (10.3)	1 (3.6)	.488
Tumour location, right, *n* (%)	36 (46.2)	15 (53.6)	.500
Tumour size before therapy (median [IQR], mm)	47.00 (32.00, 63.00)	41.50 (32.00, 66.50)	.445
Histologic type, *n* (%)			.126
Squamous cell carcinoma	38 (48.7)	15 (53.6)	
Adenocarcinoma	28 (35.9)	5 (17.8)	
Other	12 (15.4)	8 (28.6)	
cTNM stage before therapy, *n* (%)			.727
IIIB	42 (53.9)	14 (50.0)	
IIIC	36 (46.1)	14 (50.0)	
N3 lymph node metastasis, *n* (%)			**.024**
Supraclavicular	45 (57.7)	9 (32.1)	
Mediastinal or hilar	27 (34.6)	18 (64.3)	
Both	6 (7.7)	1 (3.6)	
PD-L1 TPS, *n* (%)			.118
<1%	15 (19.2)	10 (35.7)	
1-49%	15 (19.2)	8 (28.6)	
≥50%	11 (14.1)	3 (10.7)	
Unknown	37 (47.5)	7 (25.0)	
Cycles of induction therapy, median (IQR)	4.00 (3.00, 4.00)	4.00 (3.00, 4.00)	.052
Clinical response after induction therapy, *n* (%)			.196
PR	51 (65.4)	22 (78.6)	
SD	27 (34.6)	6 (21.4)	

Abbreviations: BMI, body mass index; cTNM, clinical tumour-node-metastasis; ECOG, Eastern Cooperative Oncology Group; IQR, interquartile range; PR, partial response; SD, stable disease; TPS, tumour proportion score.

a Fisher’s exact test was used.

Bold values indicate *P-*values < 0.05

**Figure 3.**
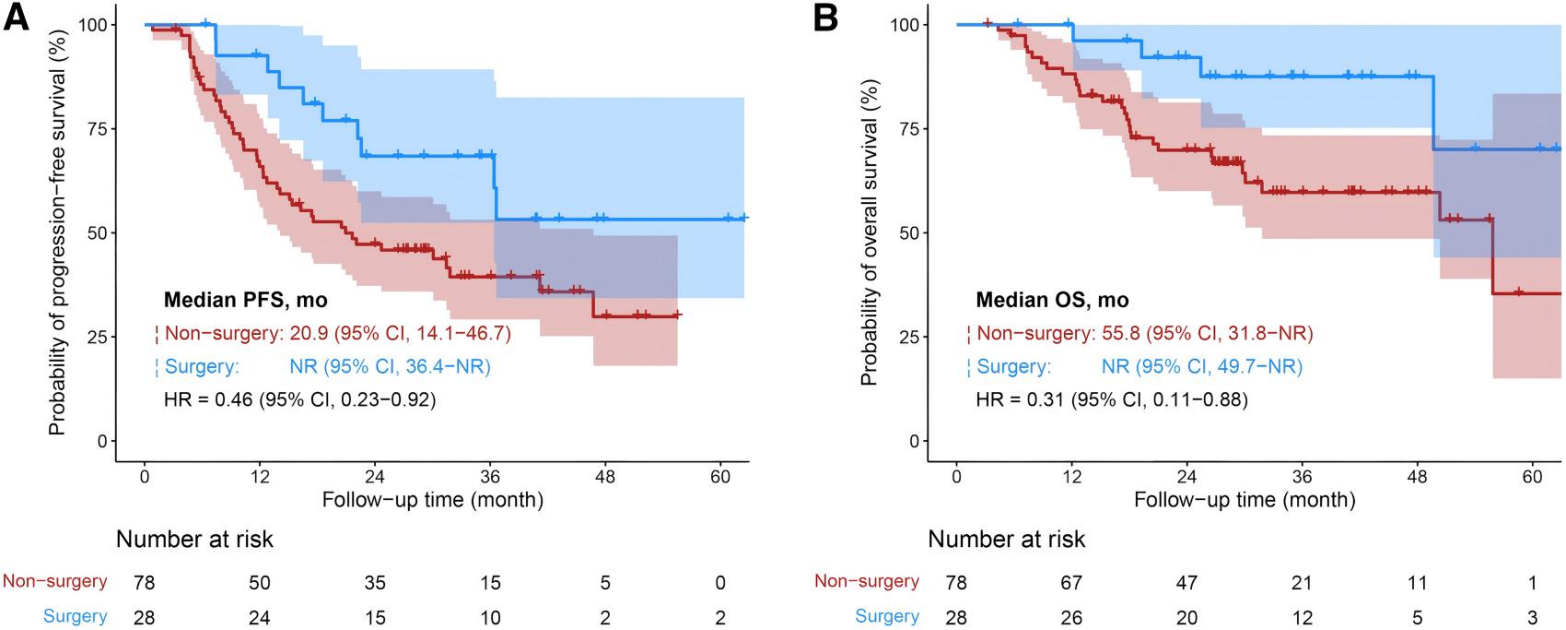
Kaplan–Meier Survival Curves of Progression-free Survival (A) and Overall Survival (B) for Surgical versus Non-Surgical Groups. Abbreviations: CI, confidence interval; HR, hazard ratio; NR, not reached; OS, overall survival; PFS, progression-free survival.

The median follow-up durations were 35.1 months (range: 6.4-68.4) in the surgical group and 33.3 months (range: 3.2-71.5) in the non-surgical group. In the surgical group, 10 (35.7%) patients had disease progression and 4 (14.3%) patients died. The median PFS and OS for the surgical group were not reached; the 2- and 4-year OS rates were 92.1% and 87.5%, and the 2- and 4-year PFS rates were 68.4% and 53.2%, respectively. In the non-surgical group, 46 (59.0%) patients had disease progression and 29 (37.2%) patients died. The median PFS was 20.9 months and median OS was 55.8 months for the non-surgical group; the 2- and 4-year OS rates were 69.9% and 59.7%, and the 2- and 4-year PFS rates were 47.2% and 29.8%, respectively. Better PFS (hazard ratio [HR] = 0.46, 95% CI, 0.23-0.92) and OS (HR = 0.31, 95% CI, 0.11-0.88) were observed in patients who underwent an operation compared to those who did not (**Figure 3A and B**).

We further analysed the surgical group by stratifying patients into those who achieved an MPR and those who did not (**Figure S4**). Notably, the MPR subgroup exhibited a 4-year OS rate of 100% and a 4-year PFS rate of 65.4%. However, the median PFS was 18.6 months and the median OS was 25.4 months for the non-MPR subgroup; the 2- and 3-year OS rates were 71.4% and 47.6%, and the 1-year PFS rate was 85.7%, respectively. Compared with non-surgical patients who achieved a partial response after induction therapy (*n* = 51), better PFS (HR = 0.36, 95% CI, 0.14-0.94) and OS (HR = 0.14, 95% CI, 0.02-1.10) were observed in patients who underwent operations and achieved an MPR (**Figure S5A and B**).

### Subgroup analysis stratified by N3 lymph node assessment method

We conducted a subgroup analysis of survival in the surgical and non-surgical groups based on different N3 lymph node diagnostic methods.

In the biopsy subgroup, 11 (20.8%) patients underwent surgical procedures, whereas 42 (79.2%) did not. The median PFS was 22.6 months and the median OS was not reached for the surgical group; the 4-year OS rate was 77.8%, and the 3-year PFS rate was 45.7%, respectively. The median PFS was 16.4 months and the median OS was 50.3 months for the non-surgical group; the 4-year OS rate was 56.3%, and the 4-year PFS rate was 27.2%, respectively. Better OS (HR = 0.64, 95% CI, 0.19-2.20) but similar PFS (HR = 0.97, 95% CI, 0.42-2.23) was observed in patients who underwent surgical procedures compared to those who did not (**Figure S6A and B**).

In the PET/CT subgroup, 17 (32.1%) patients underwent surgical procedures, whereas 36 (67.9%) did not. Neither the median PFS nor the median OS was reached for the surgical procedures group; the 4-year OS rate was 93.3%, and the 4-year PFS rate was 81.1%, respectively. The median PFS was 31.4 months and the median OS was 55.8 months for the non-surgical procedures group; the 4-year OS rate was 64.4%, and the 4-year PFS rate was 31.4%, respectively. Better PFS (HR = 0.24, 95% CI, 0.07-0.82) and OS (HR = 0.13, 95% CI, 0.02-1.01) were observed in patients who underwent surgical procedures compared to those who did not (**Figure S6C and D**).

### Landmark analysis

At 18 weeks after induction therapy, 20 (15.0%) patients were excluded from the landmark analysis due to loss to follow-up and 10 (7.5%) patients were excluded due to disease progression (**Figure S7**). Finally, there were 24 (18.1%) patients in the surgical procedures group and 79 (59.4%) patients in the non-surgical procedures group. Better PFS (HR = 0.48, 95% CI, 0.23-1.02) and OS (HR = 0.19, 95% CI, 0.05-0.81) were observed in patients who underwent surgical procedures compared to those who did not (**Figure S8A and B**).

## DISCUSSION

In this retrospective study of 133 patients with stage III-N3 NSCLC who underwent induction immuno-chemotherapy, 28 (21.1%) patients achieved N3 lymph node downstaging and underwent surgical procedures. Compared with those who did not undergo surgical procedures, patients who underwent surgical procedures showed better PFS and OS. However, In the biopsy subgroup, only OS benefits were observed in patients who underwent surgical procedures, while no PFS benefits were observed. Our findings provide early positive signals from real-world practice that surgical procedures after induction immuno-chemotherapy may be feasible in carefully selected patients with stage III-N3 NSCLC.

Given the substantial benefits of neoadjuvant immuno-chemotherapy in achieving tumour and lymph node downstaging in resectable NSCLC, current studies have gradually investigated this pattern in unresectable stage III NSCLC. In a proof-of-concept phase 2 trial, neoadjuvant SHR-1710 alone or plus chemotherapy was administered to patients with unresectable stage III NSCLC, and 27 of 107 (25.2%) patients underwent surgical procedures. Of the 43 enrolled patients with N3 disease, 9 (20.9%) underwent surgical procedures.^7^ In our study, the surgical procedures rates (21.1% vs 20.9%-40.0%) for patients with stage III-N3 NSCLC were comparable to those in previous studies.^7^^,^^19^^,^^20^ In addition, a noninferior MPR rate (75.0% vs 40.7%-58.6%) was also observed in our study compared to previous trials for stage III NSCLC.^4^^,^^5^^,^^7–9^^,^^21^

Based on survival data reported in the PACIFIC study, cCRT followed by consolidation immunotherapy has become the standard treatment for unresectable stage III NSCLC.^3^ In our study, the 2-year PFS and OS rates of 68.4% and 92.1% in the surgical procedures group were found to be numerically comparable to those from the PACIFIC study. Similarly, real-world data from 104 patients with stage III NSCLC who received surgical procedures after induction immuno-chemotherapy also exhibited similar 2-year PFS and OS rates of 61.4% and 89.9%.^20^ These findings were consistent with those from a national analysis, which demonstrated that patients with stage III-N2 NSCLC who received induction immuno-chemotherapy followed by a surgical procedure showed improved survival compared to those who received the PACIFIC regimen.^22^

The role of a surgical procedure following induction immuno-chemotherapy in patients with N3 disease remains a matter of debate.^14^ The results from 2 retrospective studies suggested that a surgical procedure after induction immuno-chemotherapy yielded better survival outcomes than non-surgical management such as maintenance immuno-chemotherapy or chemotherapy.^19^^,^^23^ Zhou et al. also indicated that a surgical procedure may offer a better prognosis compared to cCRT following immuno-chemotherapy in a phase II trial (18-month event-free survival: 74.1% vs 57.3%).^7^ Conversely, a real-world multicentre retrospective study found that an operation achieved non-optimal outcomes compared to cCRT in patients with stage III NSCLC after induction therapy.^20^ In our study, which focused on patients with stage III-N3 NSCLC, improved OS and PFS were observed in patients who underwent surgical procedures. However, the survival benefits varied among subgroups when patients were stratified by the assessment method of N3 disease. These findings imply that the apparent survival advantage in surgically treated patients after induction immuno-chemotherapy might be partly due to stage misclassification and could also partly mirror selection bias in clinical practice, where surgeons preferentially operate on younger patients with better performance status and who respond better to induction therapy.^22^

Notably, patients who underwent surgical procedures and achieved an MPR exhibited a 4-year OS rate of 100%, suggesting that surgical resection following induction immuno-chemotherapy may provide a favourable prognosis for carefully selected patients with stage III-N3 NSCLC. However, this result should be interpreted with caution, given the limited sample size and the potential instability of the estimate. Besides, this finding does not mean that the operation itself directly conferred any benefit, because an MPR is intrinsically a strong prognostic indicator.^24^ Conversely, patients in our study who underwent a surgical procedure but did not achieve an MPR exhibited a poor prognosis. Although the poor prognosis in these patients may be partly attributable to the aggressive nature of the tumour, a surgical procedure may provide local control but may concurrently risk delaying more effective systemic therapies.^20^^,^^25^

N3 lymph node metastasis was a decisive factor in defining unresectable disease in patients enrolled in this study. Despite preoperative confirmation of N3 lymph node downstaging in all surgically treated patients, approximately one-fourth were found to have a non-MPR status postoperatively, indicating that the primary tumour and metastatic lymph nodes may have had different responses in some cases.^26^ Radiologic response evaluated by CT based on RECIST v1.1 is the most commonly used method for assessing the efficacy of induction therapy.^16^ However, consistent with results from a previous study, its correlations with pathological remission and long-term survival were not always accurate.^27^ Besides, current research on immunotherapy has identified the PD-L1 expression level as a potential biomarker for response to neoadjuvant immunotherapy.^28^ Nevertheless, nearly half of the patients in this study did not undergo testing for the expression of PD-L1, and the available data could not confirm its predictive value.

This study has several limitations. First, as a retrospective study, it has a limited sample size, and selection bias is inevitable. Although 602 patients with stage III-N3 NSCLC were initially screened, only 133 met the inclusion criteria, underscoring the highly selected nature of this cohort. Second, not all patients had pathologically confirmed N3 lymph node metastasis or downstaging; reliance on PET/CT for nodal assessment introduces potential misclassification bias due to its known false-positive rate, which may impact outcome validity.^29^ Third, the decision to perform the surgical procedure was made after induction therapy. Thus, the comparison between the surgical and non-surgical groups represents a post-induction comparative analysis rather than a baseline intention-to-treat comparison. Patients selected for an operation likely possessed inherent advantages that retrospective adjustment cannot fully address, complicating causal inference.

## CONCLUSION

Our real-world data suggest that surgical resection following induction immuno-chemotherapy is feasible in a subset of carefully selected patients with stage III-N3 NSCLC. These findings provide early positive signals for the potential role of surgical procedures within a multimodality treatment strategy in this setting. However, further well-designed clinical trials are warranted to validate the role of surgical resection in the multimodality treatment of patients with stage III-N3 NSCLC.

## Supplementary Material

ivag166_Supplementary_Data

## Data Availability

All relevant data are within the manuscript and its online supplementary material.

## Abbreviations

cCRT: Concurrent chemo-radiotherapy
CI: Confidence interval
HR: Hazard ratio
IO-CT: Immuno-chemotherapy
MPR: Major pathological response
NSCLC: Non-small cell lung cancer
OS: Overall survival
pCR: Pathological complete response
PD-1: Programmed death receptor 1
PET/CT: Positron emission tomography/computed tomography
PFS: Progression-free survival
RECIST v1.1: Response Evaluation Criteria in Solid Tumours version 1.1
