# Supplementary material for: Surgery After Induction Immuno-Chemotherapy in Stage III-N3 Non-Small Cell Lung Cancer: A Single-Centre Retrospective Cohort Study
Source: Interdiscip Cardiovasc Thorac Surg. 2026 Jul 11;41(7):ivag166. doi: 10.1093/icvts/ivag166 (PMC13390708; doi:10.1093/icvts/ivag166)
Supplement: ivag166_Supplementary_Data [file ivag166_supplementary_data.docx]

| **Table S1** Surgical information of stage III-N3 NSCLC patients who underwent surgery after induction therapy | |
| --- | --- |
| **Characteristics** | **Surgery group (n=28)** |
| **Interval to surgery (median [IQR], day)** | 36.50 (31.50, 47.00) |
| **Surgical approach, n (%)** |  |
| VATS | 15 (53.6) |
| RATS | 3 (10.7) |
| Thoracotomy | 10 (35.7) |
| **Extent of resection, n (%)** |  |
| Lobectomy | 21 (75.0) |
| Bilobectomy | 1 (3.6) |
| Sleeve lobectomy | 6 (21.4) |
| **Surgical time (median [IQR], minute)** | 182.50 (123.50, 237.50) |
| **Estimated blood loss (median [IQR], mL)** | 50.00 (50.00, 100.00) |
| **Postoperative drainage time (median [IQR], day)** | 6.50 (5.00, 9.50) |
| **Length of stay (median [IQR], day)** | 6.00 (5.00, 9.00) |
| **Postoperative 30-day mortality, n (%)** | 0 (0) |
| **Postoperative 30-day morbidity, n (%)** | 15 (53.6) |
| Atrial fibrillation | 2 (7.1) |
| Pneumonia | 3 (10.7) |
| Pleural effusion | 9 (32.1) |
| Prolonged air leak | 7 (25.0) |
| **Clavien-Dindo grade, n (%)** |  |
| 0 | 13 (46.4) |
| 1-2 | 12 (42.9) |
| ≥3 | 3 (10.7) |
| **Re-intervention, n (%)** | 3 (10.7) |
| **Examined lymph node, median [IQR]** | 16.00 (11.00, 25.00) |
| **Positive lymph node, median [IQR]** | 0 (0, 0) |

*IQR*: interquartile range; *VATS*: video-assisted thoracoscopic surgery; *RATS*: robotic-assisted thoracoscopic surgery.

| **Table S2** The treatment information of stage III-N3 NSCLC patients who underwent surgery after induction therapy | |
| --- | --- |
| **Treatment type** | **Surgery group (n=28)** |
| **Adjuvant therapy, n (%)** |  |
| Immuno-chemotherapy | 11 (39.3) |
| Chemotherapy | 2 (7.1) |
| Immunotherapy | 4 (14.3) |
| No | 11 (39.3) |
| **Radiotherapy, n (%)** | 5 (17.9) |

| **Table S3** The treatment information of stage III-N3 NSCLC patients who did not undergo surgery after induction therapy | |
| --- | --- |
| **Treatment type** | **Non-surgery group (n=78)** |
| **Treatment after induction therapy, n (%)** |  |
| Chemoradiotherapy | 32 (41.0) |
| Maintenance immunotherapy | 34 (43.6) |
| Others | 12 (15.4) |

**SUPPLEMENTARY** **FIGURE LIST**

**
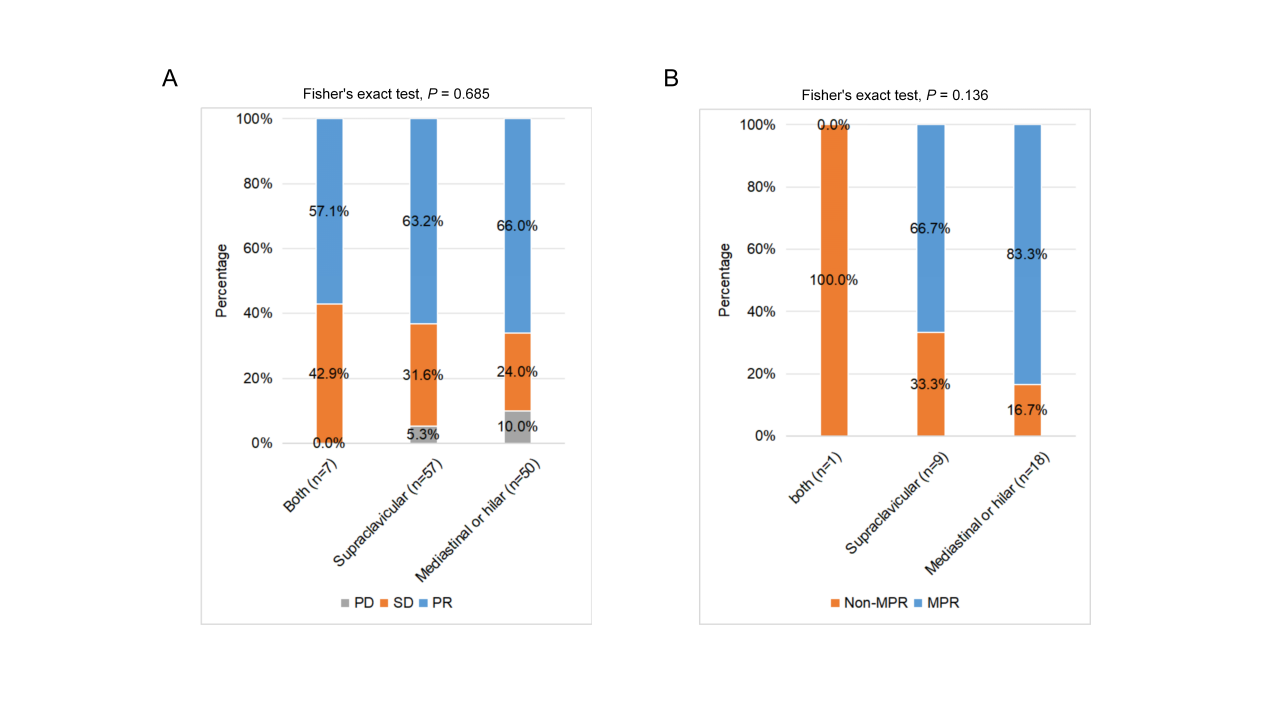
**

**Figure S1.** The efficacy of induction therapy (A) and the pathological response status (B), stratified by the metastatic site of N3 lymph nodes. *PR*: partial response; *SD*: stable disease; *PD*, progressive disease; *MPR*, major pathological response.

**
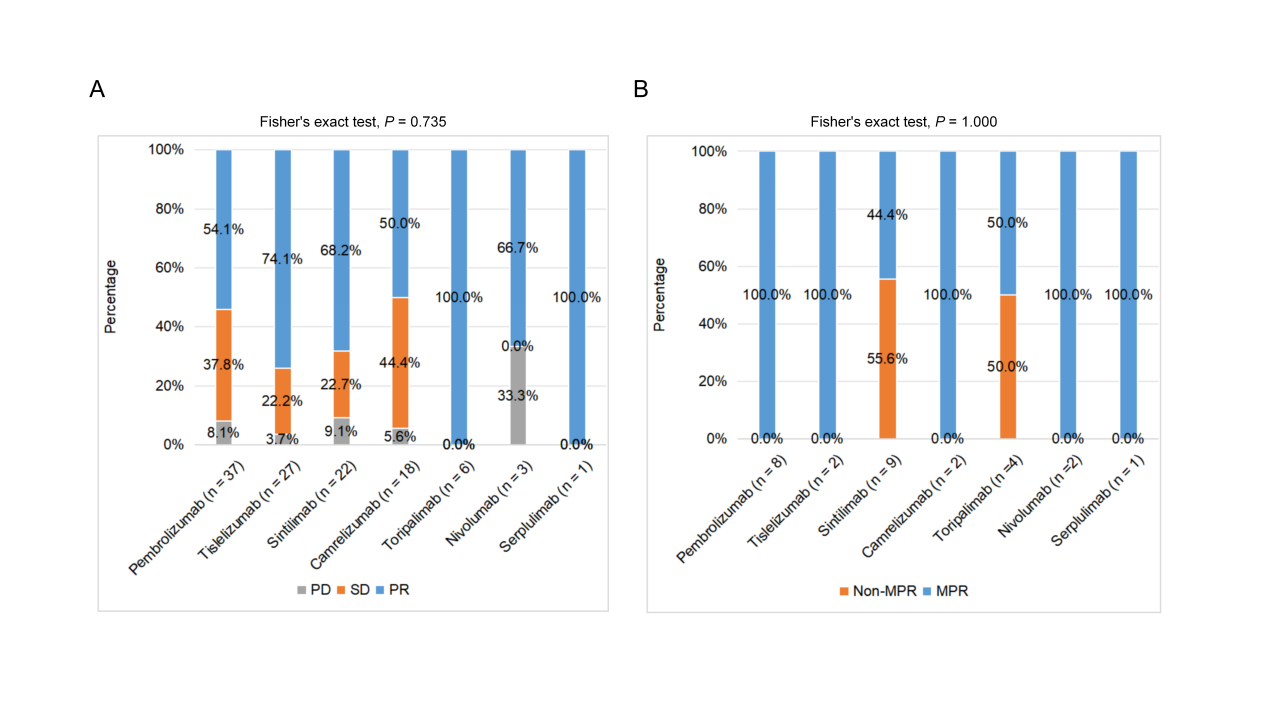
**

**Figure S2.** The efficacy of induction therapy (A) and the pathological response status (B), stratified by the type of PD-1 blockade. *PR*: partial response; *SD*: stable disease; *PD*, progressive disease; *MPR*, major pathological response.


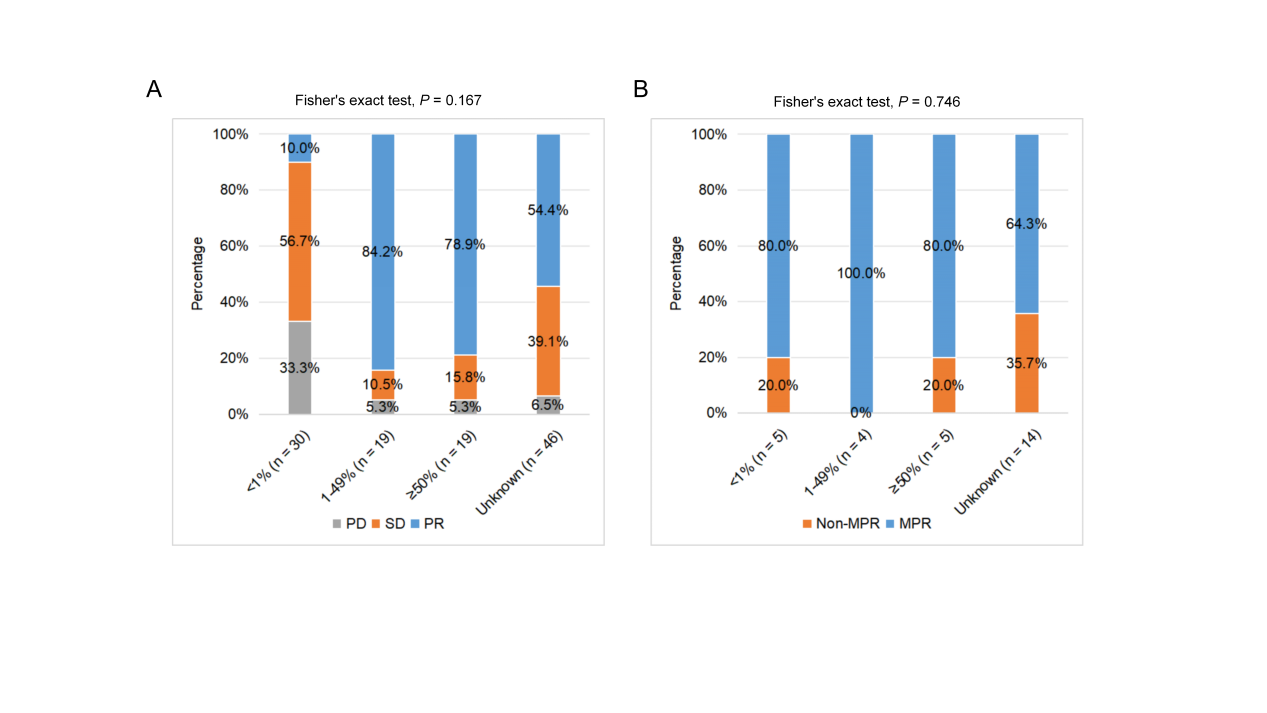


**Figure S3.** The efficacy of induction therapy (A) and the pathological response status (B), stratified by PD-L1 expression levels. *PR*: partial response; *SD*: stable disease; *PD*, progressive disease; *MPR*, major pathological response.


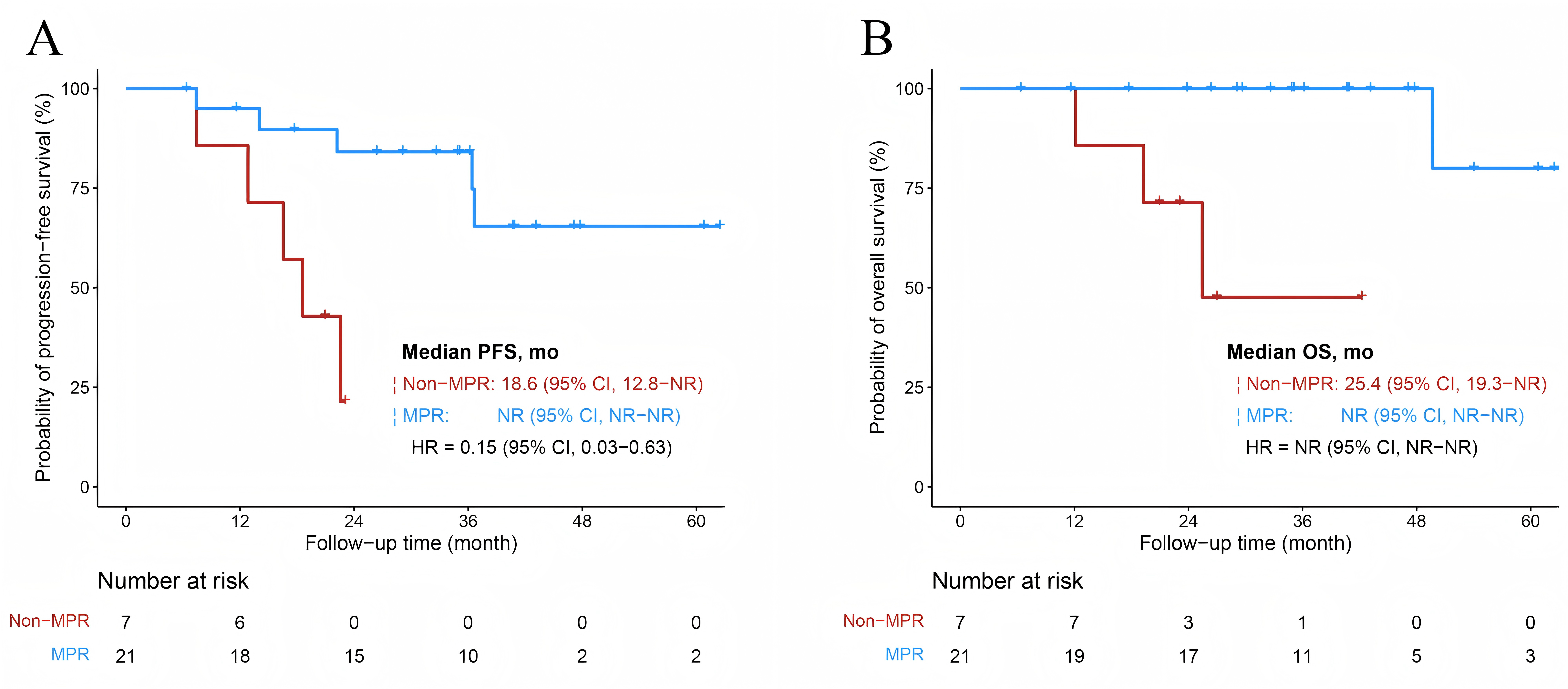


**Figure S4.** Kaplan-Meier survival curves of PFS (A) and OS (B) for MPR vs. non-MPR groups. *MPR*, major pathological response; *PFS*, progression-free survival; *OS*, overall survival; *HR*, hazard ratio; *CI*, confidence interval; *NR*, not reached.


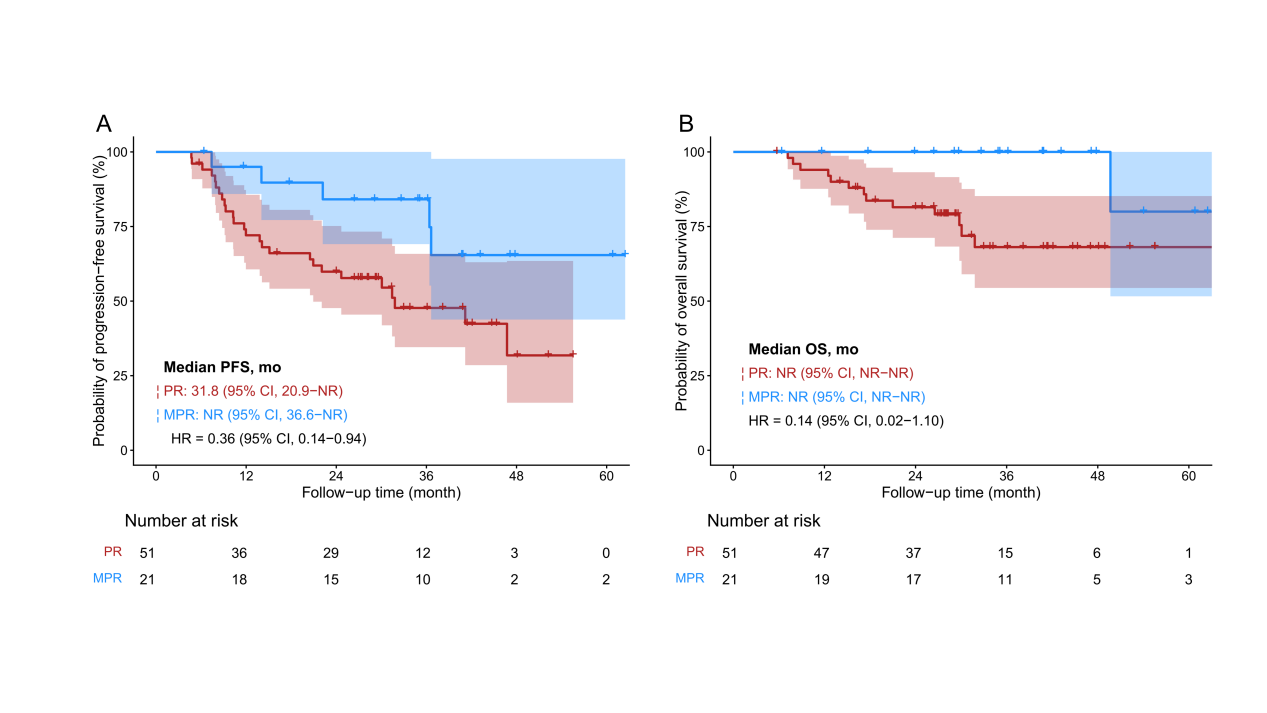


**Figure S5.** Kaplan-Meier survival curves of PFS (A) and OS (B) for MPR vs. PR groups. *MPR*, major pathological response; *PR*, partial response; *PFS*, progression-free survival; *OS*, overall survival; *HR*, hazard ratio; *CI*, confidence interval; *NR*, not reached.


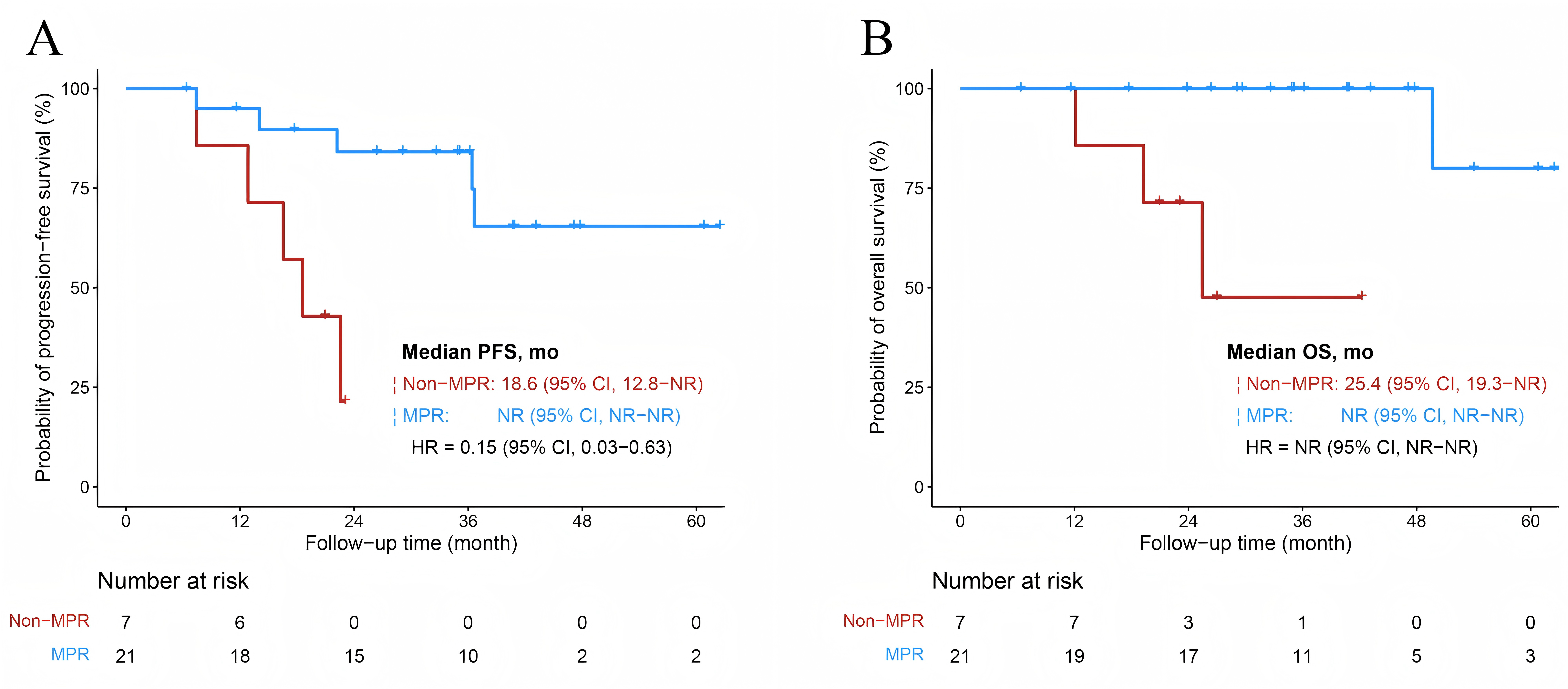


**Figure S6.** Kaplan-Meier survival curves of PFS and OS for surgery vs. non-surgery groups stratified by N3 lymph node assessment method. (A) Comparison of PFS in the biopsy subgroup. (B) Comparison of OS in the biopsy subgroup. (C) Comparison of PFS in the PET/CT subgroup.(D) Comparison of OS in the PET/CT subgroup. *PFS*, progression-free survival; *OS*, overall survival; *HR*, hazard ratio; *CI*, confidence interval; *NR*, not reached.


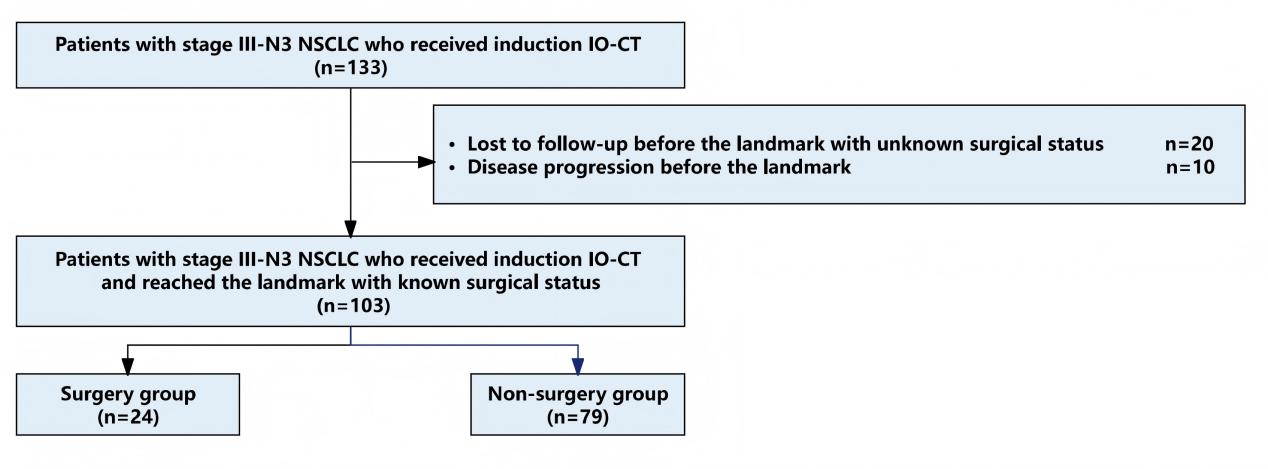


**Figure S7.** Flowchart for landmark analysis. *NSCLC*, non-small cell lung cancer; *IO-CT*, immuno-chemotherapy.


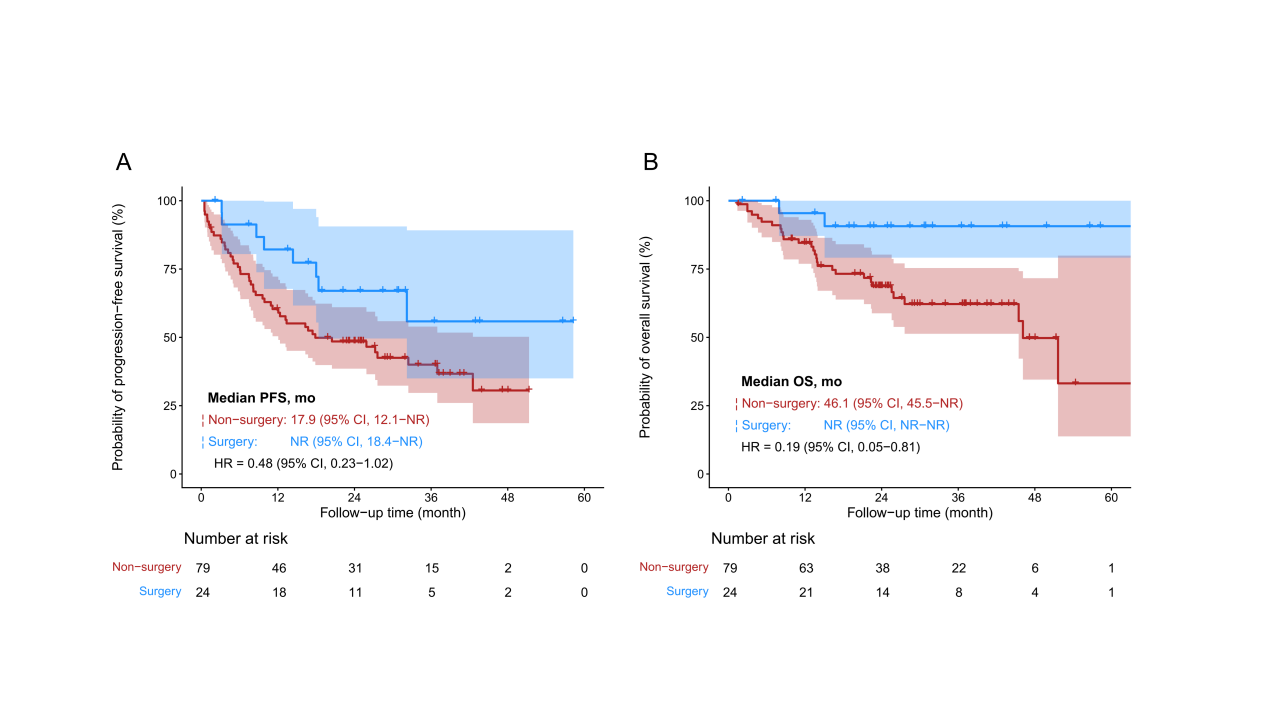


**Figure S8.** Kaplan-Meier survival curves of PFS (A) and OS (B) for surgery vs. non-surgery groups in the landmark analysis. *PFS*, progression-free survival; *OS*, overall survival; *HR*, hazard ratio; *CI*, confidence interval; *NR*, not reached.
